# Evaluating knowledge, habits, and beliefs regarding dietary supplements as a protective measure against COVID‐19 in Malaysia and Iraq: A postsecond wave cross‐sectional analysis

**DOI:** 10.1002/hsr2.1865

**Published:** 2024-02-09

**Authors:** Ali Haider Mohammed, Bassam Abdul Rasool Hassan, Ali Blebil, Juman Dujaili, Abdulrasool M. Wayyes, Osama Ayad Abdulhamid, Humam Saadi Salih, Watheq Mohammed AL‐Jewari, Hawar Sardar Hassan, Angelina Lim

**Affiliations:** ^1^ School of Pharmacy Monash University Malaysia Bandar Sunway Malaysia; ^2^ Department of Pharmacy Al Rafidain University College Baghdad Iraq; ^3^ Swansea University Medical School Swansea University Swansea UK; ^4^ Department of Dentistry Komar University of Science and Technology Kurdistan‐Region Iraq; ^5^ Department of Radiology Anwar‐Sheikha Medical City, Sulaimani Kurdistan Region Iraq; ^6^ Faculty of Pharmacy and Pharmaceutical Sciences Monash University Parkville Victoria Australia; ^7^ Murdoch Children's Research Institute Royal Children's Hospital Parkville Victoria Australia

**Keywords:** beliefs, COVID‐19, dietary supplement, habits, knowledge

## Abstract

**Background and Aims:**

The Corona Virus Disease 2019 (COVID‐19) pandemic brought to the forefront various public health approaches, including the consumption of dietary supplements (DS) as a protective measure. With misinformation regarding the virus and the associated benefits of DS prevalent, this study aimed to understand knowledge, habits, and beliefs related to DS usage as a protective measure during the pandemic in Malaysia and Iraq, two countries with deep‐rooted traditions in herbal and supplement usage.

**Methods:**

A cross‐sectional research study was conducted between September 2021 and March 2022 using a validated online survey. The participants included Malaysians and Iraqis aged 18 years and above who currently consume DS. Using the SurveyMonkey® platform, data were collected from 2425 respondents (response rate = 60.6%), with analysis carried out using SPSS version 28.

**Results:**

Demographically, the sample had an almost equal distribution of Malaysians (51%) and Iraqis (49%), with a mean age of 30.61. The majority had tertiary education (78.6%), and only a fraction had been infected with COVID‐19 (26.2%). Concerning knowledge, a significant portion exhibited poor understanding (84.2%) of DS's functioning and implications. Regarding habits, many respondents consumed multivitamins (75.2%), with influence largely coming from peers (23.5%) and product leaflets (46.7%). Belief‐wise, about half (49.2%) utilized herbal or supplemental products as a protective measure during the pandemic, with vitamin C with zinc being the most commonly used (45.4%).

**Conclusion:**

The study underlines a significant inclination towards DS usage in Malaysia and Iraq, influenced by societal connections and available information. While many believe in the protective capacities of DS against COVID‐19, a substantial knowledge gap persists. It emphasizes the need for evidence‐based awareness campaigns and policies to guide public health decisions.

## 1 INTRODUCTION

Dietary supplements (DS) refer to the different products intended for ingestion that contain a certain concentration of nutrients, which would enhance the usual diet. DSs in different forms, such as powder, capsules, liquids, and even bars, which would be loaded with the specified nutrients. A previous literature study reported that the consumption of DSs is driven by either personal motivation or external influence with the common goal of improving their health.^1^ Several people have started consuming DSs as a personal choice to improve their personal health. People usually consume DSs either when they feel that they have a deficiency in their diet or when they perceive that having an increment of certain nutrients would help them desire their achieved results, such as losing weight, having clearer skin, increased energy, and athletic performance as documented by Karbownick et al.^2^ Consequently, people would search for the best method in which they can enhance their current diets to have an increased measure of the anticipated nutrients. On the other hand, people are also driven to consume DS through external influences such as advertising and recommendations from athletic coaches, health practitioners, and even friends. Additional influences include social media and reviews from the brand ambassadors who would have been engaged to advocate for the consumption of these brands.^3^ In this instance, the users would not actively look for the DS but would be influenced to consume them due to the information shared on the benefits of consuming these supplements. While the consumption of DS has been considered as an additional element of conventional treatment methods, it has been gradually rising to prominence as a mainstream method of disease prevention and treatment as evidenced in the Corona Virus Disease 2019 (COVID‐19) pandemic.^3^

The impact of the COVID‐19 pandemic is significant in various nations across the world, which have called for a variety of methods to curb the spread of the virus. People have been generally proactive in taking steps to prevent infection and treat the infection effectively. While this is a noble cause and reflective of the collaborative efforts in providing public healthcare solutions, it has also opened up another dimension of the case of wrong information concerning the prevention and treatment of the virus.^4^ During the COVID‐19 pandemic, there has been an increased interest and use of DS by individuals. The reasons for this heightened consumption can be attributed to several factors. First, there is a perception that certain DS ingredients can boost immune function and reduce inflammation, which may help prevent COVID‐19. Many people are seeking ways to strengthen their immune system and protect themselves against the virus. Second, the pandemic has caused widespread anxiety and stress, and some individuals may turn to DS as a means of supporting their mental health and well‐being. DS containing vitamins and minerals are often associated with promoting overall health and vitality, which may be appealing to individuals during this challenging time. Finally, there is a growing concern for general health and wellness, and individuals may view DS as a way to supplement their diet and address any nutrient deficiencies that could compromise their immune system.^5^ The World Health Organization has been constantly making efforts to spread information regarding the virus, which includes tips on how to prevent infection and treat the virus. However, there have also been other alternative influences that have been providing often conflicting information over the treatment of this virus.^6^ One of the major issues that has emerged is the usage of health supplements as a protective measure during the COVID‐19 pandemic. Health supplements were typically used as a means to boost immunity during the pandemic and also gained popularity as a treatment regimen for patients who had been affected by the virus.^7^ While this can be regarded as a positive factor and proactive method of preventing and treating the virus, it raises multiple public health concerns. One of the major issues is that the efficacy of DS has been overestimated and misrepresented, which reflects how they can often be deemed as inappropriate as a protective measure against COVID‐19.^8^ Moreover, the overuse of DS can lead to several disadvantages, including the potential for drug–dietary supplement interactions. These interactions can occur when DS interfere with the effectiveness or safety of prescription medications, leading to decreased therapeutic effects or adverse events. For example, interactions between tetracyclines (a type of antibiotic) and calcium or magnesium supplements can reduce the absorption of the antibiotic, affecting its effectiveness. In addition, overuse of DS can result in an excess intake of certain nutrients, leading to side effects such as gastrointestinal disturbances, organ damage, or toxicity. It is important for individuals to be aware of these potential interactions and side effects, and to consult with healthcare professionals before starting any DS regimen to ensure safe and appropriate use.^9^ There is ongoing research exploring the potential use of DS and nutraceuticals in managing the long‐term effects of COVID‐19. These supplements include amino acids like glutamine and branched‐chain amino acids, which may support immune response and overall health. Other potential options include hydroxy‐beta‐methylbutyrate, tricarboxylic acid cycle intermediates, and micronutrients such as selenium and zinc.

Adequate nutrition, particularly for older adults who may be at a higher risk of malnutrition, is important in mitigating the long‐term effects of COVID‐19. Nutritional interventions, including the consumption of bioactive foods, supplements, and nutraceuticals, may play a role in managing these symptoms.^10^

It is essential to investigate the knowledge and habits regarding health supplements and beliefs about the usage of health supplements as a protective measure during the COVID‐19 pandemic in Malaysia and Iraq for varied reasons. First, both of these countries produce a wide array of supplements that address health and wellness issues.^11^, ^12^, ^13^, ^14^ Therefore, the consumption of DS is an existing phenomenon that has been readily accepted. These countries are also renowned for the usage of herbs and having alternative treatments from conventionally prescribed medicine. However, there are limited studies that address the usage of DS in such countries, which have already been grounded in the use of herbs and supplements, which this study seeks to address. An additional reason that reflects the importance of this study is the issue of the pandemic, where it is vital to measure how such countries that are already used to the usage of supplements would react to the pandemic. There are high chances that these countries have prescribed alternative methods for the prevention and treatment of the COVID‐19 virus thus it is necessary to investigate the current phenomenon of DS usage in light of the pandemic. Thus, the current study aimed to evaluate the level of knowledge and habits about DS and belief about the usage of the health supplements as a protective measure during the COVID‐19 pandemic in Malaysia and Iraq as a representative of middle‐income countries.

## 2 METHODS

### 2.1 Study design and population

Cross‐sectional research was conducted in Malaysia and Iraq between September 2021 and March 2022 using a validated and reliable online survey. Malaysia and Iraq were selected as a sample of Asian and Arab populations, respectively, as their societies have various cultures, habits, and beliefs^12^, ^13^ so that a general insight about their people's knowledge, habits, and beliefs toward DS can be formed. The study population consisted of: (i) Malaysians who are particularly residing in Malaysia and Iraqis who are residing only in Iraq and their aged 18 years and above; and (ii) currently taking a DS including vitamins, minerals, and other less familiar substances; as herbals, amino acids, and enzymes.^15^ Exclusion criteria included refusal to participate in the study and cognitive impairments that affect the ability to think, read, or concentrate. The sample size for this study was predicated on the hypothesis that half of the participants would respond to the principal questions, as response rates in Malaysia and Iraq had not been previously established. The sample size calculation utilized the Raosoft® sample size calculator with a 5% margin of error, a 95% confidence level, and an anticipated 50% response rate.^16^ Thus, the minimum sample size estimated for the study was 385.

### 2.2 Study instrument and translation

The survey instrument was developed based on extensive literature reviews on DS.^2^, ^17^, ^18^ The questionnaire was reviewed by three researchers to assess its appropriateness, relevance, clarity, and sufficiency. It included 48 questions, divided into sociodemographic characteristics (nine questions), knowledge about DS (17 true/false items), habits related to food supplementation (six questions), and beliefs regarding the use of herbal products and/or food supplements as a protective measure against COVID‐19 (16 items). Initially composed in English, the survey was subsequently translated into Bahasa Malaysia and Arabic by a certified translator. A reverse translation process was employed to ensure accuracy. The forward translation was performed by translators with specific sector knowledge and experience, with Bahasa Malaysia and Arabic as their mother tongues. A second translator, a native English speaker, then back‐translated the questionnaire into English. The back translator was deliberately chosen to have no professional or personal ties to the initial translator. The authors conducted a comparative review between the original and the back‐translated questionnaires, identifying and classifying discrepancies as minor, such as wording issues, or significant, such as grammatical errors or alterations in meaning. The translations underwent a final review by two experts to ensure accuracy and fidelity of meaning. Discrepancies were resolved through consensus in a meeting with the authors and translators, where one coauthor documented all queries and comments. Preliminary testing on 20 individuals assessed content, design, readability, and comprehension, leading to further refinements for clarity and precision. The questionnaire's validity and reliability were confirmed through a pilot study involving 30 DS consumer adults, yielding Cronbach's *α* values of 0.89 and 0.94 for knowledge items and 0.93 and 0.97 for belief items, indicating high reliability and internal consistency.

### 2.3 Data collection

Data was gathered online via SurveyMonkey® from September 2021 to March 2022. Participants were recruited anonymously through convenience sampling and were solicited via social media. The participants were asked to give their consent before filling up the questionnaire by being asked to click the “agree” button if they were willing to participate in the study. To maximize reach within the two countries, the study employed multiple dissemination strategies, involving research assistants, the researchers' professional and personal networks, community leaders, and social media influencers. The survey was primarily shared through Facebook®, Twitter®, Instagram®, and WhatsApp®, with Facebook® and WhatsApp® being particularly popular among the Arab population, and Twitter® and Instagram® among the younger demographic. A standard description of the survey preceded the questionnaire link in WhatsApp messages and social media posts, in both Malay and Arabic. Out of 4000 invited participants (approximately 2000 from each country), 2425 consented to participate and completed the survey, resulting in a response rate of 60.6%. All collected data were anonymized and maintained confidentially.

### 2.4 Data analysis

Data analysis was conducted using SPSS version 28 (IBM Corp). Categorical data were summarized as frequencies and percentages, and continuous data as means with standard deviations. The Kolmogorov–Smirnov and Shapiro–Wilk tests confirmed the normal distribution of the data. Knowledge levels regarding DS were quantified using a scoring system, with a continuous variable derived from the total correct answers to 17 questions. Each correct response earned one point, with no points for incorrect answers. Knowledge scores were adapted and modified from previous literature studies^2^, ^17^, ^18^ and then classified into three levels: poor (0–6), average (7–12), and good (13–17). Previous literature studies^2^, ^7^, ^18^ provided a foundation for these classifications. These studies have analyzed data that suggested natural breaks in the distribution of scores, indicating where the boundaries between poor, average, and good might lie. Besides, there are also theoretical reasons for choosing these intervals. For example, our questionnaire included 17 questions, then a score of 13 or above could be considered good because it is above 75% of the total points, which is a commonly used cutoff for “good” performance.

Differences in mean knowledge scores among demographic groups were analyzed using the *T* test and one‐way analysis of variance, with HSD post hoc tests identifying sources of significant variation between groups. A 5% significance level and a 95% confidence interval (*p* < 0.05) were the thresholds for statistical significance. Multivariate regression analysis was conducted to identify independent predictors of participants' knowledge, utilizing sociodemographic characteristics and levels of knowledge as independent variables. This analysis aimed to discern the individual contributions of various factors to the overall understanding held by study participants.

## 3 RESULTS

### 3.1 Demographic characteristics of participants

Table 1 demonstrated that the majority of the respondents (51%) were Malaysians while the other respondents (49.0) were of Iraqi descent. The mean age of the respondents was 30.61 and the females were more presented than the males (54.3%). Most of the respondents indicated that they had reached tertiary level education (78.6%) while only a small proportion of the respondents (1.6%) indicated that they were uneducated. In terms of health levels; the respondents largely indicated that they were healthy (54.1%) and only about a quarter (26.2%) had been previously infected with COVID‐19 before. Despite this, nearly a third of the respondents indicated that they were athletic and just over a 10th indicated that they have a chronic disease (12.5%). The sociodemographic characteristics of the respondents are presented in Table 1.

**Table 1:** Sociodemographic characteristics of respondents (*n* = 2425).

Characteristic	Frequency (%)
Country	
Malaysia	1237 (51.0)
Iraq	1188 (49.0)
Gender	
Male	1108 (45.7)
Female	1317 (54.3)
Ethnicity	
Malay	182 (7.5)
Malaysian Chinese	950 (39.2)
Arabs	1088 (44.8)
Others	205 (8.5)
Educational level	
Uneducated	37 (1.5)
Primary	145 (6.0)
Secondary	337 (13.9)
Tertiary	1906 (78.6)
Are you athletic	
Yes	741 (30.6)
No	1684 (69.4)
Do you have any chronic disease?	
Yes	303 (12.5)
No	2122 (87.5)
Were you infected with COVID‐19 before?	
Yes	636 (26.2)
No	1789 (73.8)
How do you rate your health?	
Healthy	1313 (54.1)
Slightly healthy	951 (39.2)
Unhealthy	161 (6.7)
Mean	Standard deviation
Age	
30.61	23.343

### 3.2 Participants' knowledge toward DS

The participants in this study were queried on their knowledge regarding their general and intrinsic knowledge about DS (Table 2). The respondents indicated overall poor knowledge (84.2%) of how DS work and interact with various health issues. The respondents were mostly wrong on the notion that DS are food; where only 34.8% of the respondents were able to respond correctly to this question. The respondents were mostly correct on the notion that taking excessive amounts of magnesium supplements can cause diarrhea and nausea as 82.5% of the respondents answered this question correctly. The respondents also reflected the conflicting issues in society, as just over half of the respondents (55.2%) agreed that chemicals can be sold both as medicine and as a supplement. The respondents indicated very little knowledge as only a few respondents managed to correctly answer questions on issues of the efficacy and safety of DS (4.5%); the packaging of DS (7.7%) and the interaction of calcium supplements and bone structure (9.1%).

**Table 2:** Participants' knowledge about dietary supplements.

Items	Correct answer (true [T] or false [F])	Frequency (%)
*7 items general*		
Before being marketed, dietary supplements must be tested for efficacy and safety	F	110 (4.5)
A chemical ingredient may be sold both as a medicine and as a dietary supplement	T	1339 (55.2)
The quality of dietary supplements is routinely tested before being marketed	F	3.0 (12.7)
The packaging of dietary supplements must contain information on possible adverse effects resulting from their use	F	186 (7.7)
Dietary supplements are food	T	844 (34.8)
Dietary supplement registration requires assessing the composition of the product by the appropriate supervisory body	F	233 (9.6)
All dietary supplements sold in pharmacies have been tested for safety	F	516 (21.3)
*10 items specific*		
Taking vitamin and mineral supplements prevents diseases in healthy people	F	436 (18.0)
In the elderly, taking vitamin D reduces the risk of bone fractures	F	333 (13.7)
In the elderly, the use of magnesium preparations prevents muscle cramps	F	564 (23.3)
Taking dietary supplements containing calcium reduces the risk of bone fractures in the elderly	F	221 (9.1)
The use of multivitamin preparations protects against heart diseases	F	933 (38.5)
The use of antioxidants prevents the development of cancer	F	779 (32.1)
Regular use of vitamin C prevents the risk of catching a cold	F	378 (15.6)
Taking excessive amounts of magnesium supplements can cause diarrhea and nausea	T	2000 (82.5)
Vitamin C naturally present in food is better assimilated than synthetic	F	427 (17.6)
People with kidney disease should not use high doses of vitamin C	T	1836 (75.7)
Level of knowledge (mean ± standard deviation: 4.72 ± 1.97)		
Poor	2043 (84.2%)	
Average	374 (15.4%)	
Good	8 (0.4%)	

### 3.3 Participants' habits related to food supplementation

**Figure 1:**
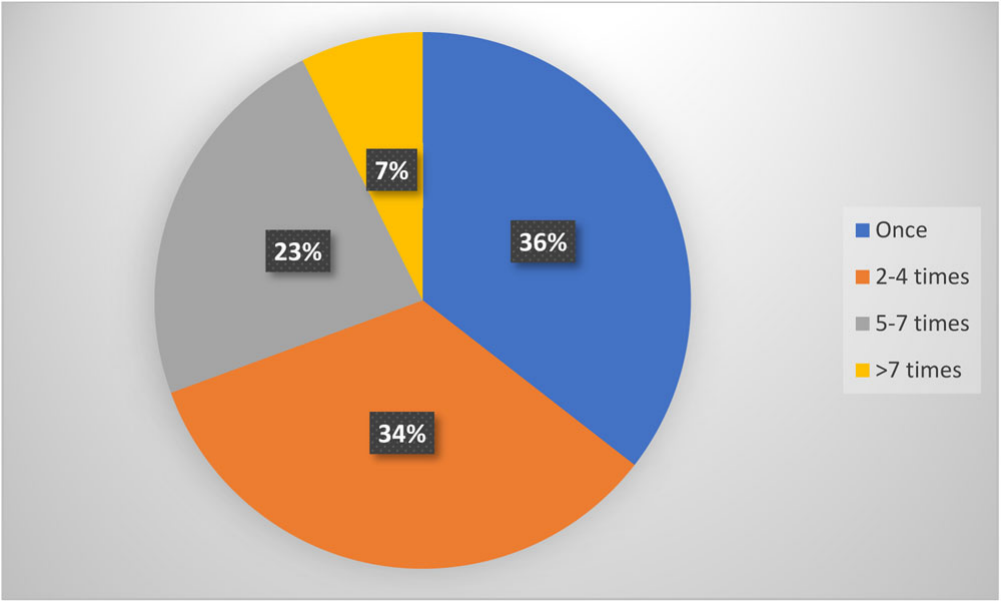
Participants' times of using dietary supplements per week.

The participants were asked about their habits related to food supplementation and about a quarter stated that they use DS 5–7 times a week (23%) while just over a third indicated that they use the supplements only once a week (36%) (Figure 1).

Nevertheless, almost all of the respondents have consumed multivitamins (75.2%) followed by sports drinks (24.2%) and amino acids (22.1%) respectively, as shown in Table 3. The respondents were largely influenced by friends and coworkers (23.5%) to use food supplements while only 11% were influenced by media advertising. The respondents largely indicated that the use of DS was influenced by the perception of physical weakness (39.6%) and at times just randomly (30.4%). Information of the product on the leaflet was indicated as one of the most common influencers of DS usage (46.7%). While over half of the respondents stated not experiencing any side effects (75.5%); nausea and vomiting was stated as the most common side effect experienced by the users (7.8%).

**Table 3:** Participants' habits about dietary supplements.

Items	Variables	Frequency (%)
What types of food supplements have you consumed? (more than one answer is allowed)	Branched‐chain amino acids, protein powder, and creatine	536 (22.1)
	Multivitamins	1823 (75.2)
	Sport drinks	587 (24.2)
	Weight‐loss products	260 (10.7)
	Stimulants (caffeine pills, guarana, and ginseng)	260 (10.7)
	Others	275 (11.3)
Who influenced your decision to use food supplements? (more than one answer is allowed)	No one	710 (29.3)
	Trainer/coach	174 (7.2)
	Friend/coworker	571 (23.5)
	Medical doctor	558 (23.0)
	Pharmacist	412 (17.0)
	Parents/relatives	541 (22.3)
	Internet	551 (22.7)
	Media advertising	267 (11.0)
Under what circumstances have you taken food supplements?	In association with sports activity	420 (17.3)
	Periodically throughout the year (e.g., in association with season changes)	305 (12.6)
	When I perceive physical weakness	961 (39.6)
	Sporadically, without any particular pattern	738 (30.4)
How have you established the amount of food supplement you should take? (More than one answer is allowed)	Based on a seller's advice	598 (24.7)
	Based on a trainer/coach's advice	326 (13.4)
	Based on an indication in the product information leaflet	1133 (46.7)
	Found information on internet	597 (24.6)
	Calculated based on body weight	285 (11.8)
	Arbitrarily	348 (14.4)
What side effects (if any) have you experienced whilst taking food supplements? (More than one answer is allowed)	None	1830 (75.5)
	Nausea/vomiting	188 (7.8)
	Diarrhea	170 (7.0)
	Constipation	11 (4.6)
	Abdominal pain	152 (6.3)
	Headache	181 (7.5)
	Others	92 (3.8)

### 3.4 Participants' beliefs about DS as a protective measure against COVID‐19

**Figure 2:**
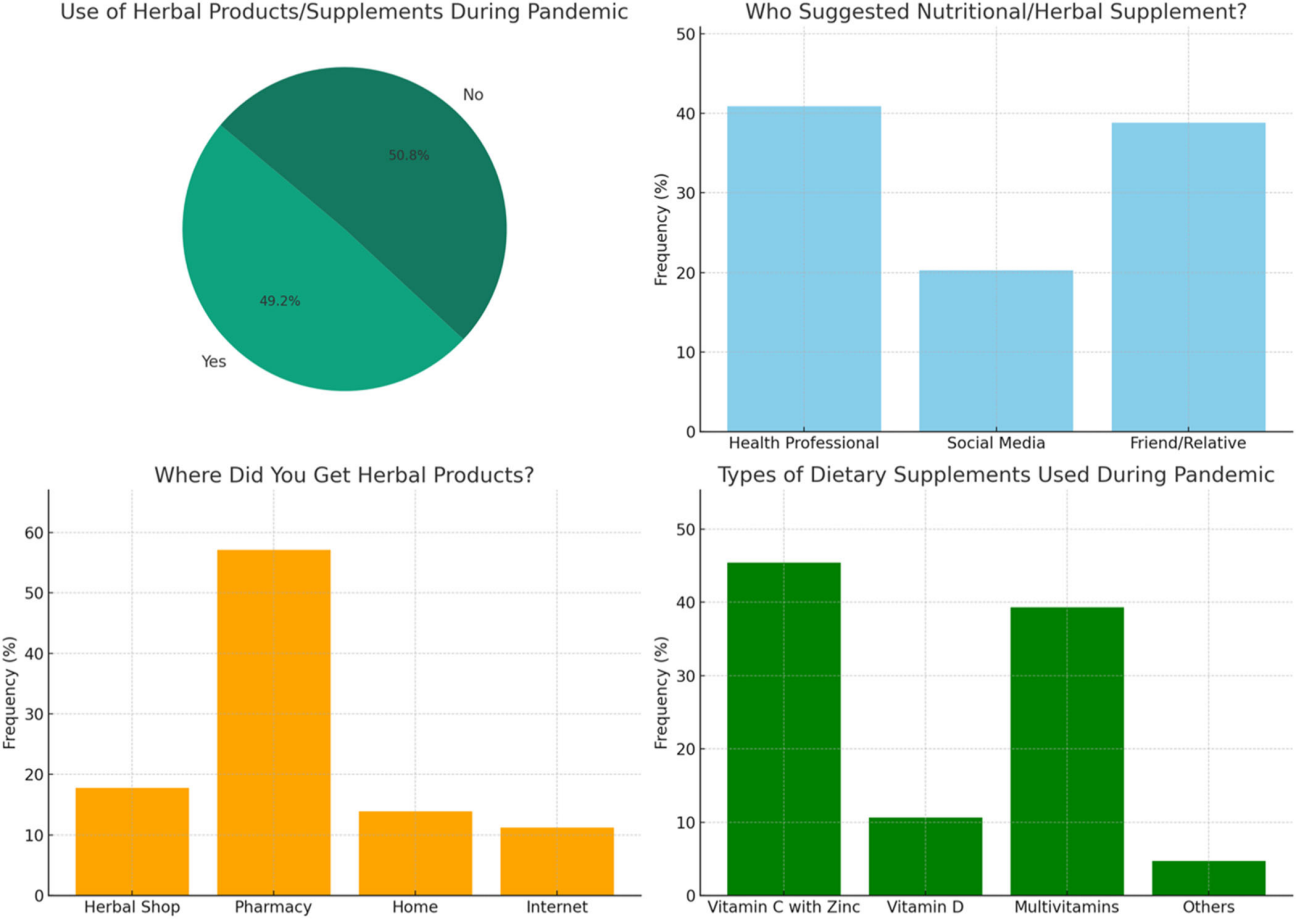
Belief about the use of the herbal products and/or food supplements as protective measure against COVID‐19.

The participants' belief about DS as a protective measure against COVID‐19 was also probed (Figure 2). Nearly half of the respondents used herbal products or supplements as a protective measure during the pandemic (49.2%). The most common recommendation for using DS came from dieticians and physicians (40.9%) followed by recommendations from friends and relatives (30.8%). The most common DS the respondents used before the onset of the pandemic is vitamin C with zinc (45.4%) and over half of the respondents acquired these supplements from a pharmacy (57.1%).

Furthermore, the participants were also interviewed on their beliefs about the use of herbal supplements and food supplements as a protective measure against COVID‐19 (Table 4). The results are presented in Table 4. The respondents largely agreed that consumption of vitamin C found in citrus has a role in treating or reducing the chances of developing COVID‐19 (45.2%), vitamins and herbal supplements treat/reduce the incidence of COVID‐19 (38.9%) and that eating garlic helps to increase the immunity and reduce the chance of developing COVID‐19 (38.9%). The respondents also largely refused that applying sesame oil on the body protects from COVID‐19 (53.9%) and that consuming of nutritional and herbal supplements prevents the spread of COVID‐19 more than social distance (43.0%). The respondents also indicated a lack of knowledge that taking ginseng extract tablets helps to increase the immunity and protect us from corona infection (35.6%).

**Table 4:** Belief about the use of the herbal products and/or food supplements as protective measure against COVID‐19 (*n* = 2425).

Items	Yes	No	Maybe	I do not know
Do you think that drinking turmeric tea helps increase immunity and reduce the chance of developing COVID‐19?	454 (18.7)	610 (25.2)	758 (31.3)	603 (24.9)
Do you think ginger tea helps to increase the immunity and reduce the chance of developing COVID‐19?	560 (23.1)	574 (23.7)	765 (31.5)	526 (21.7)
Do you think that eating garlic helps to increase the immunity and reduce the chance of developing COVID‐19?	944 (38.9)	515 (21.2)	662 (27.3)	304 (12.5)
Do you think that eating onions (or onion peel) helps to increase the immunity and reduce the chance of developing COVID‐19?	786 (32.4)	608 (25.1)	665 (27.4)	366 (15.1)
Do you think that eating fish oil known as omega‐3 helps to increase the immunity and reduce the chance of developing COVID‐19?	873 (36.0)	427 (17.6)	779 (32.1)	346 (14.3)
Do you think that taking ginseng extract tablets helps to increase the immunity and protect us from corona infection?	347 (14.3)	511 (21.1)	703 (29.0)	864 (35.6)
Do you think that consumption of vitamin C found in citrus has a role in treating or reducing the chances of developing COVID‐19?	1097 (45.2)	308 (12.7)	706 (29.1)	314 (12.9)
Do you think that vinegar plays a role in treating or protecting against COVID‐19?	242 (10.0)	922 (38.0)	569 (23.5)	692 (28.5)
Do you think salted water plays a role in treating or protecting against COVID‐19?	379 (15.6)	921 (38.0)	554 (22.8)	571 (23.5)
Do you think that applying sesame oil to the body protects from COVID‐19?	135 (5.6)	1307 (53.9)	399 (16.5)	584 (24.1)
Do you think that consuming nutritional and herbal supplements prevents the spread of COVID‐19 more than social distancing?	405 (16.7)	1043 (43.0)	670 (27.6)	307 (12.7)
Do you think that vitamins and herbal supplements treat/reduce the incidence of COVID‐19?	944 (38.9)	347 (14.3)	944 (38.9)	190 (7.8)

### 3.5 Association between demographic characteristic and participants' knowledge

In the analysis of participant knowledge scores, several sociodemographic factors exhibited statistically significant differences (Table 5). Knowledge scores varied slightly between countries, with participants from Malaysia scoring marginally higher on average (4.73) than those from Iraq (4.71), indicating a statistically significant difference (*p* = 0.019). Gender differences were pronounced and significant, with male participants scoring higher (4.95) compared to females (4.52, *p* < 0.001). Ethnicity also played a role, with “others” scoring the highest (5.08), suggesting substantial variability in knowledge across ethnic groups (*p* = 0.017). Educational attainment correlated significantly with knowledge scores; surprisingly, uneducated participants scored the highest (5.76), which may warrant further investigation (*p* = 0.003). Athletic individuals did not differ significantly from nonathletic ones in terms of knowledge scores (*p* = 0.275). There was no significant difference in knowledge scores based on the presence of chronic disease (*p* = 0.658). Participants who had not been infected with COVID‐19 had lower knowledge scores than those who had been infected (4.65 vs. 4.90, *p* = 0.029), potentially reflecting an information‐seeking behavior postinfection. Moreover, overall health status was a significant predictor of knowledge, with unhealthy participants scoring considerably higher (5.63), which could suggest a heightened search for health‐related information in this group (*p* < 0.001). Lastly, the use of herbal products or nutritional supplements as a protective measure against COVID‐19 was associated with lower knowledge scores (4.42 for users vs. 5.0 for nonusers, *p* < 0.001), indicating a potential gap in evidence‐based understanding among users.

**Table 5:** Differences between knowledge score with participants' demographic characteristics.

Characteristics	Knowledge score mean	*p* Value
Country^a^		0.019*
Malaysia	4.73	
Iraq	4.71	
Gender^a^		<0.001*
Male	4.95	
Female	4.52	
Ethnicity^b^		0.017*
Malay	4.74	
Chinese	4.75	
Arabs	4.62	
Others	5.08	
Education level^b^		0.003*
Uneducated	5.76	
Primary	4.82	
Secondary	4.87	
Tertiary	4.66	
Athletic^a^		0.275
Yes	4.86	
No	4.65	
Chronic disease^a^		0.658
Yes	4.71	
No	4.71	
Infected with COVID‐19^a^		0.029*
Yes	4.90	
No	4.65	
Overall health^b^		<0.001*
Healthy	4.64	
Slightly healthy	4.67	
Unhealthy	5.63	
Using herbal products or nutritional supplements to protect against COVID‐19^a^		<0.001*
Yes	4.42	
No	5.0	

^a^ Independent *t* test.

^b^ One‐way analysis of variance test.

* Statistically significant value (*p* < 0.05).

### 3.6 Independent predictors for participants' knowledge

In this multivariate regression analysis, the reference categories for each categorical variable are specified, with coefficients indicating the expected change in the dependent variable when switching from the reference category to the other categories, controlling for all other factors as reported in Table 6. Being from Iraq is associated with a decrease of 0.1712 points compared to Malaysia, and this effect is statistically significant (*p* = 0.045). Female respondents are expected to score 0.0922 points higher than males, with a significant *p* value of 0.030. When comparing ethnicity, being Chinese, Arab, or Other as opposed to Malay is associated with an incremental increase of 0.0611 points in the score, which is statistically significant (*p* = 0.040). Higher educational levels, particularly reaching tertiary education, are strongly associated with an increase in scores by 0.3194 points, highly significant with a *p* value of less than 0.001. The athletic variable, however, is not statistically significant (*p* = 0.247), suggesting no significant difference in scores between those who are athletic and those who are not. Having no chronic disease is related to an increase in scores by 0.1383 points, which is significant (*p* = 0.020). Those not infected with COVID‐19 are expected to have a 0.1769 point increase in scores compared to those who were infected, showing a significant relationship (*p* = 0.035). Finally, moving from a healthy to a slightly healthy or unhealthy status is associated with a minor and marginally significant increase in scores by 0.0489 points (*p* = 0.050).

**Table 6:** Independent predictors of participants' knowledge toward dietary supplements.

Variables	Coefficient	*p* Value*
Country	−0.1712	0.045
Malaysia (Ref)		
Iraq		
Gender	0.0922	0.030
Male (Ref)		
Female		
Ethnicity	0.0611	0.040
Malay (Ref)		
Chinese		
Arabs		
Others		
Education level	0.3194	<0.001
Uneducated (Ref)		
Primary		
Secondary		
Tertiary		
Athletic	−0.1189	0.247
Yes (Ref)		
No		
Chronic disease	0.1383	0.020
Yes (Ref)		
No		
Infected with COVID‐19	0.1769	0.035
Yes (Ref)		
No		
Overall health	0.0489	0.050
Healthy (Ref)		
Slightly healthy		
Unhealthy		

* Statistically significant *p* < 0.05.

## 4 DISCUSSION

DS has been highly consumed, especially in high‐ and middle‐income countries. Thus, there is an urgent need to understand public knowledge, habits, and beliefs about the usage of DS among the middle‐income population. This is because to develop a comprehensive program and educational intervention to reduce the risk of unwanted side effects from inappropriate use of DS. To the best of our knowledge, this study is the first of its kind to address such a critical issue among populations with various cultures and habits. This study finds that DS are used widely among Malaysian and Iraqi populations by consuming wide categories of health supplements that might promise great benefits to the users, such as improved skin, stronger nails, longer hair, weight loss, and even stronger bones, other than addressing serious health concerns. These promises are endearing to the potential user, which lures many people into buying these supplements and expecting great results despite the limited evidence that supports these claims. This is consistent with the findings of previous studies, which reported that advertising campaigns are often compelling and are designed to gain the trust and confidence of the user, who would anticipate quick and effective results that would address their needs.^19^, ^20^ Therefore, this has driven users to regard supplements as highly effective in addressing their health needs regardless of limited evidence.

Based on the study findings, the observed differences in knowledge scores between participants from Iraq and Malaysia could be due to a variety of factors that are rooted in the sociodemographic and cultural contexts of the two countries. First, the small but statistically significant difference in knowledge scores, with Malaysian participants scoring slightly higher on average than those from Iraq, might be influenced by the educational systems and the availability of health information in each country.^16^ The dissemination of knowledge about health and DS may be more robust in one country due to better health education initiatives, public health campaigns, or more accessible health information through media and public health institutions. Second, cultural factors could play a significant role. In Malaysia, there might be a greater emphasis on traditional herbal knowledge and natural remedies as part of the cultural heritage, which could contribute to a higher awareness and knowledge of DS.^5^, ^8^, ^13^ On the other hand, Iraq has its own rich tradition of herbal remedies and natural medicine, but the impact of recent conflicts and political instability may have affected the public health infrastructure and education, potentially leading to differences in health knowledge dissemination. Finally, the influence of social networks and peer education should not be underestimated. In some societies, knowledge about health and supplements is often passed through social and family networks. Differences in social structures and the extent of these informal knowledge networks between Malaysia and Iraq could contribute to the knowledge gap observed.^15^, ^18^

Moreover, this study reported that DS has been used by people for reasons other than health concerns but rather aesthetics, which alludes to the wider perception of these supplements. For example, instead of engaging in a healthy diet and exercise, some users would prefer to take weight loss supplements despite the lack of any results from such a practice.^21^, ^22^ This could partly explain why people perceive supplements as a shortcut and instant fix that can produce the desired results.^23^ Therefore, there are some people who might deem the prescribed COVID‐19 prevention measures such as social distancing and personal hygiene and hope that despite practicing these; they would be unscathed by the pandemic as long as they take their supplements.

DS were easily accepted as prevention against COVID‐19 due to their popularity among the population. DS has been previously used as part of a healthy lifestyle regimen where people would take different supplements as they perceived fit for their body. The study of Rondanelli et al. reflects how there is a wide range of DS that have been used, with vitamin C and zinc ranking as part of the most popular food supplements that are being used.^24^ These findings are further reflected in the study of Thomas et al. and Hoang et al. supplements have been associated with the prevention and treatment of the common cold, and this is one of the possible factors why this DS was quickly adopted in the pandemic.^25^, ^26^

People generally overestimate the efficacy of DS in addressing their health needs in general. This study finds that there are beliefs that regular usage of vitamin C would prevent the common cold and of calcium would prevent fractured bones. While these claims are actually false, they point toward the exaggerated perception that customers have of the efficacy of these supplements. While these studies have suggested a potential positive effect of administering vitamin C and zinc supplements for the prevention of infection from COVID‐19, these claims are debunked in the study of Hemilä and Chalker, which states negligible differences in patients before and after taking such supplements.^27^

Trust and confidence in the efficacy of DS as a preventive measure against COVID‐19 has been noted, though its origins are still unclear. A possible cause would be that people have been reliant on herbal products and medicine over the years; thus, they would regard the DS as an extension of the herbal products.^28^, ^29^ This study finds a significant relationship between ethnicity and the knowledge of DS, with the Malaysians and Chinese showing higher levels of knowledge. This is rationalized by how these ethnicities have been using herbal products over the years, and these remedies are now being currently incorporated into the treatment of COVID‐19. These findings are supported by studies that have extensively investigated the integration of Chinese herbal medicine in the prevention and treatment of COVID‐19 and have indicated slightly positive results.^30^, ^31^, ^32^, ^33^

The influence of social media in prescribing preventive treatments against COVID‐19 cannot be undermined. Due to the nature of social media, it is easy for information to spread quickly, despite its quality and accuracy.^33^ Therefore, the prescription of DS to bolster one's immunity against COVID‐19 was quickly spread despite the lack of any fundamental findings that would back this claim. This is reflected in the study of Qc, Sissung and Figg, and Wierzejska et al., where there are several cases of advertising claims which were on social media where the usage of supplements was presented as effective in curbing COVID‐19.^34^, ^35^, ^36^

This study finds that the habits of people towards the usage of DS are spread through the influence of friends or relatives rather than qualified personnel. This indicates why the usage of these DS is often hard to control because of the source it stems from. This supports the notion of Bykic et al. that when people do not have sufficient information to give medical advice, they should desist from it as it would have stronger implications in the long run.^37^ The respondents in this study indicated that they would opt for DS when they perceive weakness, which is consistent with the increased reliance on these supplements during the pandemic. However, this is contrasted by the study of Rao et al. and Puścion‐Jakubik et al. where the high mortality rate in the pandemic paired with the overburdening of the healthcare system prompted people to resort to seeking out DS despite not feeling weak at all.^38^, ^39^ Therefore, the usage of DS could serve as a reflection of the limitations of the healthcare system to adequately support people during the pandemic.

While the usage of supplements might be heavily promoted, it is essential to note that there are limited scientific backings that render them effective. There is very little information that links the tangible results of improved health to the usage of the various DS.^40^ In fact, there are studies that have probed into the potentially toxic ingredients and repercussions in both DS and herbal products.^41^ Despite these limited findings; people still continually use the DS and it can be noted that this suggests that people lack sufficient knowledge of how DS work. In this study, it is noted that people might have limited knowledge of the safety protocol which supplements should undergo before they are fit for usage; which poses the threat of exposing the public to unsafe ingredients. The lack of proper knowledge concerning DS also tallies with lower education levels, where people with lower levels of knowledge and exposure to health issues are more likely to believe the unfounded claims of DS effective in the prevention and treatment of diseases.^42^, ^43^, ^44^ Additionally, this study finds that people have an erroneous perception of the abilities and limitations of the DS; indicating expectations of avoiding bone structures and preventing cancer. This erroneous belief seems to be compounded by the advertising of the DS which promise innumerable benefits to the users despite sufficient evidence.

The present study's findings regarding DS habits during the pandemic highlight a significant reliance on multivitamins, sports drinks, and amino acids among participants. This pattern of DS consumption aligns with behaviors observed in other developing countries, like Indonesia and Malaysia, where there has been a notable increase in supplement use during the COVID‐19 pandemic, often influenced by social media and personal contacts rather than medical advice.^45^, ^46^

The substantial increase in DS usage during the second wave of COVID‐19 in our study could be attributed to heightened concerns over health due to the rising case numbers, similar to trends seen in Indonesia. However, unlike the Indonesian study, which did not specifically correlate DS consumption with COVID‐19 case numbers, our study suggests that the prevalence of DS use may be a response to the perceived threat and uncertainty of the pandemic's second wave.^45^

In terms of vaccination coverage, the Indonesian study's timeframe only included vaccinations for healthcare professionals, the elderly, and public servants. In contrast, our study's period may have encompassed a broader vaccination rollout, though specific comparisons of vaccination coverage rates were not within our study's scope.^45^ The Malaysian study, conducted around the same time, reported a 41.9% prevalence of herbal and dietary supplements (HDS) usage for COVID‐19 prevention, highlighting the influence of age and prior HDS usage as predictors, which might suggest a possible relationship between vaccination availability and the perceived need for additional preventive measures.^46^

The reliance on friends and coworkers for DS information, as reported by our respondents, parallels the Malaysian findings, where respondents similarly obtained HDS information from nonprofessional sources. This indicates a common pattern across countries that may impact public health initiatives, emphasizing the need for credible, accessible information on DS usage and its implications.^46^

The occurrence of side effects, such as nausea and vomiting reported by our participants, was relatively low. This is consistent with the Malaysian study, which also expressed concerns about the concurrent use of HDS with conventional medications. It suggests a need for healthcare providers to be proactive in educating the public about the safe use of DS, particularly during a pandemic when individuals may be more inclined to self‐medicate.^46^

The aggressive marketing of DS also promoted their wide usage in the pandemic. While there are few to none dietary supplementary brands that claimed to help users prevent infection from COVID‐19; it can be noted that their promotion was rather aggressive in the pandemic. These advertisements claimed that the supplements would help fight symptoms of the common cold; which are highly similar to those of COVID‐19. As a result, people deemed these supplements as appropriate alternatives that would increase their levels of immunity while also lowering their chances of having an infection, yet this has been found to be fraudulent in previous literature.^47^, ^48^, ^49^ These studies indicate that the advertising of DS is not entirely true and can be termed as misleading, thus it cannot be relied upon as a source of information.

## 5 LIMITATION

The study has several limitations that should be considered when interpreting the findings. First, relying on self‐reported data introduces the possibility of recall bias and social desirability bias. The absence of a control group makes it challenging to establish a causal relationship between DS usage and perceived benefits or beliefs so that future study might consider to conduct a similar study with a control group. Moreover, multivariate analysis can be considered as well in the future study.

## 6 CONCLUSION

This study has revealed a gap in the understanding of DS among participants, where the impact of peers and social media on supplement‐related behaviors is marked, frequently leading to the adoption of medically unsubstantiated practices. To mitigate this, we advocate for more stringent regulation of DS promotion and advertising, ensuring that claims of benefits are evidence‐based and not misleading. Enhanced public education on the proper use and realistic effects of DS is crucial, as it will aid consumers in making informed decisions about their intake, particularly in relation to their health and well‐being. Furthermore, accurately communicating the potential and limitations of DS is imperative for supporting public health initiatives, including those aimed at controlling the COVID‐19 pandemic effectively.

## AUTHOR CONTRIBUTIONS

All authors have read and approved the final version of the manuscript. Ali Haider Mohammed had full access to all of the data in this study and takes complete responsibility for the integrity of the data and the accuracy of the data analysis.

## CONFLICT OF INTEREST STATEMENT

The authors declare no conflict of interest.

## ETHICS STATEMENT

This study was conducted according to the guidelines laid down in the Declaration of Helsinki and all procedures involving research study participants were approved by the Monash University Human Research Ethics Committee and the Institutional Research Ethics Committee of Al Rafidain University College granted the ethical approval for this study (MUHREC‐30419; EC‐72‐2021), respectively. Written consent was obtained from all individual participants in the study. Participants signed informed consent regarding publishing their deidentified data.

## TRANSPARENCY STATEMENT

The lead author Ali Haider Mohammed affirms that this manuscript is an honest, accurate, and transparent account of the study being reported; that no important aspects of the study have been omitted; and that any discrepancies from the study as planned (and, if relevant, registered) have been explained.

## Data Availability

The data that support the findings of this study are available from the corresponding author upon reasonable request.
